# Not as random: the stable dynamics controlling shallow convective clouds

**DOI:** 10.1038/s41612-025-00924-1

**Published:** 2025-02-07

**Authors:** Ilan Koren, Tom Dror, Elizabeth-Ruth Shehter, Orit Altaratz

**Affiliations:** Department of Earth and Planetary Sciences, Weizmann Institute of Sciences, Rehovot, Israel

**Keywords:** Climate sciences, Atmospheric science, Hydrology

## Abstract

Shallow, sparse, non-precipitating convective clouds forming over the ocean are considered among the least organized cloud fields. The formation mechanism of these clouds is associated with random, local perturbations that create buoyant parcels. Their sparseness suggests no or very weak interactions between clouds. Here, we show that such clouds form within a well-organized, stable, dense mesh of convective cells that operate continuously, independent of the presence of visible clouds.

Shallow convective clouds play a crucial role in the climate system by reflecting part of the solar radiation back to space while emitting in the longwave at a temperature close to the Earth’s surface. This reflective property results in a net cooling effect. Moreover, such clouds couple the boundary layer and the free atmosphere, transferring aerosol, heat, moisture, and momentum across the layers and preconditioning the atmosphere for deeper clouds^1,2^.

The typical size of shallow convective clouds is much smaller than the grid boxes of operational global climate models. Therefore, such clouds are not explicitly resolved but parameterized as sub-grid processes, complicating the accurate representation of their properties and trends. Knowledge gaps regarding the basic properties of shallow convective cloud fields and their expected feedback to global warming contribute significantly to the uncertainties in climate projections^1,3^.

The organization of clouds within the field is an important factor in determining their overall properties^4,5^. The spatial and temporal organization properties reflect nonlinear processes that affect the field. The emergence of cloud organization suggests “communication” between the dynamical building blocks of the field^6^ that, via feedback loops, produce regular patterns that can be remarkably stable over time^4,7^. These patterns indicate that the underlying convective cells are not locally independent phenomena but rather a structured field, exhibiting a collective behavior^7,8^. The collective behavior affects the cloud’s lifetime, size, albedo, and rain patterns^6^.

Small-sized, non-precipitating convective clouds forming over the trade winds region of the subtropical oceans are regarded among the most unorganized cloud patterns^9^. These Trade-Cumulus (TrCu) clouds are often referred to as “sugar” cloud fields because they resemble small white grains scattered randomly over the dark ocean. Sugar-TrCu have the smallest and least dense clouds, resulting in a relatively low overall cloud fraction^10^. They are thought to form through a process often described as a local thermal perturbation near the ocean’s surface within the atmospheric boundary layer. This disturbance can generate a buoyant parcel that cools as it ascends, potentially causing the supersaturation level to exceed a critical value and form a cloud.

Clouds form a complex, multi-scale system where dynamic, thermodynamic, chemical, and radiative processes interact across various temporal and spatial scales^11^. Here, we focus on the cloud field scale (10s to 100s km) and on the convective elements within the field, which serve as its dynamic building blocks (ranging from 100s m to 10s km). We explore the properties of these supposedly unorganized cloud fields utilizing both geostationary satellite observations and a detailed cloud-resolving model (see the “Methods” section). Shallow cloud fields are drifted by the wind. As an initial step, we have developed a Lagrangian approach to track cloud fields and to correct for their drift^7^ (see “Methods” for details). Within the Lagrangian framework, we follow the evolution of key properties of the field (reflectance in satellite data and vertical velocity in the model’s output), projecting the data into a Lagrangian Hovmöller space (LHS). At each time step, a selected line (trace) is copied from the corresponding Lagrangian corrected snapshot (of satellite image or model output) and pasted as a column in the LHS. The LHS provides a spatiotemporal description of the trace’s evolution^4^. Features sampled by the trace (clouds or updrafts) will be depicted as horizontal lines, where the line’s vertical extent reflects the size of the feature, as sampled by the trace, and its horizontal length indicates the feature’s lifetime.

Figure 1 shows a 5-min interval reflectance-LHS analysis of a “sugar” type shallow, sparse convective cloud fields observed by the GOES-16 satellite^12^ over the Caribbean Sea on Feb 6, 2018. The typical lifetime of a sugar cloud is ${\mathcal{O}}(10)$ minutes. The LHS (Fig. 1, panels b, c, and d) shows continuous horizontal lines composed of multiple bright segments, persisting for time intervals ranging from a few minutes to tens of minutes. Notably, the segments tend to align along continuous lines, even if slightly tilted, due to imperfections in the Lagrangian corrections. This pattern suggests that a steady, continuous convective process might drive cloud formation. Specifically, if the same convective cell operates persistently and, after the Lagrangian correction, remains approximately stationary, clouds forming and dissipating over the cell will produce segments that align along a continuous line in the LHS. Such behavior contrasts the anticipated characteristics of a cloud field governed by local random perturbations.

**Fig. 1:**
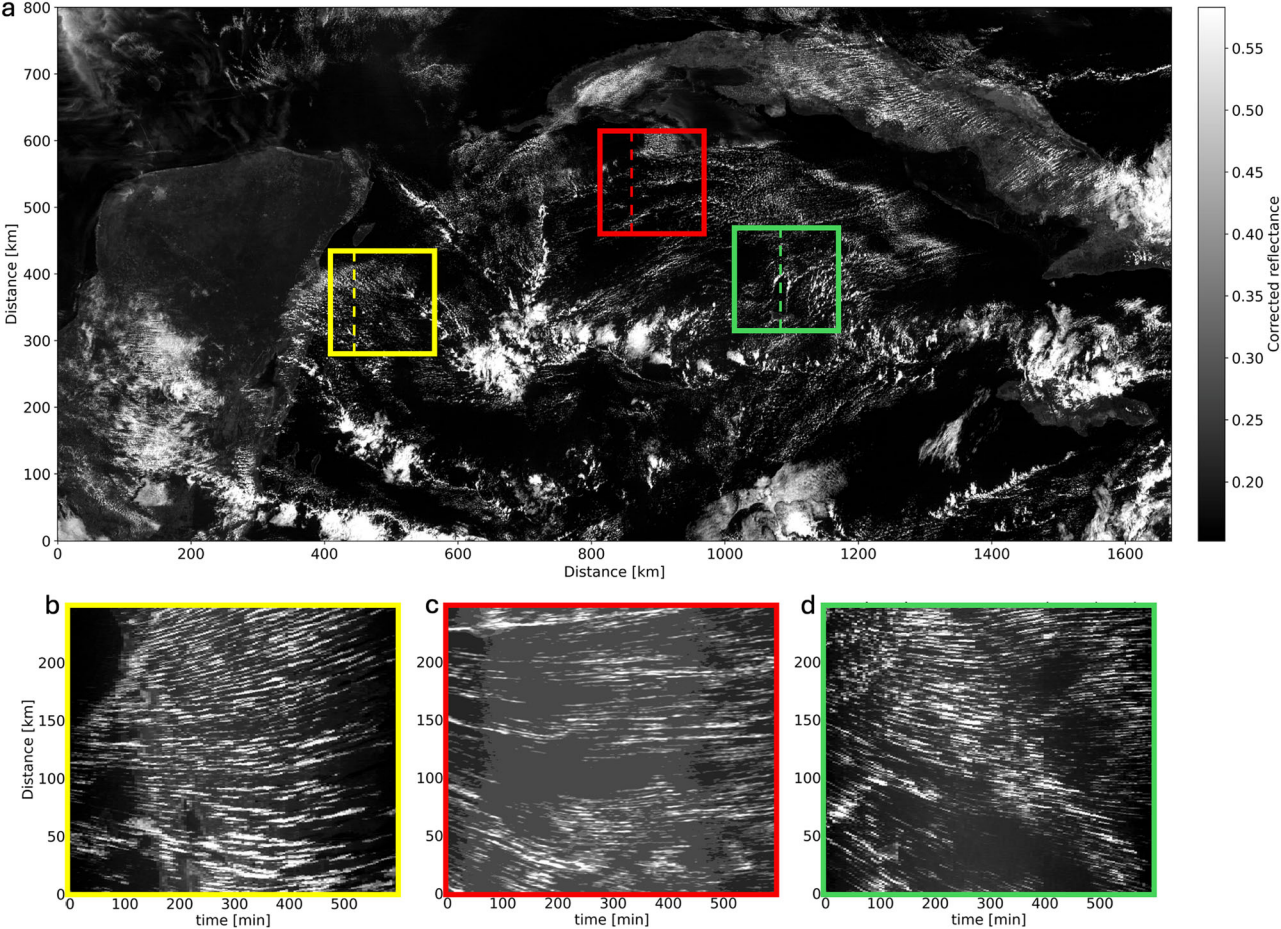
Lagrangian Hovmöller space analysis of sugar Trade Cumulus (TrCu) clouds. **a** GOES--16 satellite image taken over the Caribbean Sea on February 6, 2018. The yellow, red, and green polygons mark fields of non-precipitating small convective TrCu clouds. The dashed lines in each polygon indicate the location of the vertical trace extracted from each Lagrangian corrected image to form the LHS shown in (**b**, **c**, **d**). Each clear white segment in the panels represents a cloud. The thickness of the segment is proportional to the sampled cloud size (along the selected trace), while the segment length represents the cloud lifetime. Note the continuity of the lines, where many new segments appear along the same trajectory as previous clouds.

To better understand the dynamical mechanisms underlying such fields, we have simulated a non-precipitating, sugar-type TrCu field using state-of-the-art large-eddy simulation (LES) model^13^ coupled with a Spectral Bin Microphysical (SBM) scheme^14^. LES models serve as the main tool for creating realistic cloud fields. The simulation outputs a 4D field of all critical dynamical variables contributing to the formation of the cloud field. Figure 2a shows a snapshot of the updraft (*w*) field below the cloud base. Rising air (updraft) is a necessary condition for forming convective clouds. The *w*-field fully occupies the domain, creating cellular structures where updrafts are located along the cell boundaries while downdrafts reside at the cell center. Such a structure is typically associated with open-cells in stratocumulus (Sc) cloud decks^15,16^, which are governed by precipitation-driven oscillations^17,18^. Such a recharge-discharge cycle^4,19^ completely differs from the non-precipitating, small, and sparse TrCu clouds analyzed here. Fig. 2b shows a 1-min interval *w*-LHS analysis along the yellow trace marked in Fig. 2a. The *w*-LHS analysis indicates that the partition of updrafts and downdrafts along the trace is remarkably stable. Many cells exist continuously throughout the 6 h of the simulation. As pointed out above, updraft locations mark places where convective clouds can form. When the convective cell’s structure is almost fixed in time, for many hours, the locations of the clouds should follow it. To explore this hypothesis, we marked the location of each cloud centroid (see “Methods”) in every 1-min snapshot after Lagrangian correction. Fig. 2c shows the cloud-centroid locations accumulated over a 3-h time period, starting at the time of the updraft snapshot in Fig. 2a. The non-random cloud-centroid spatial distribution reveals a clear partition into cloud-free zones surrounded by cloud-permitting ones, mirroring the cellular structure of the updraft snapshot.

**Fig. 2:**
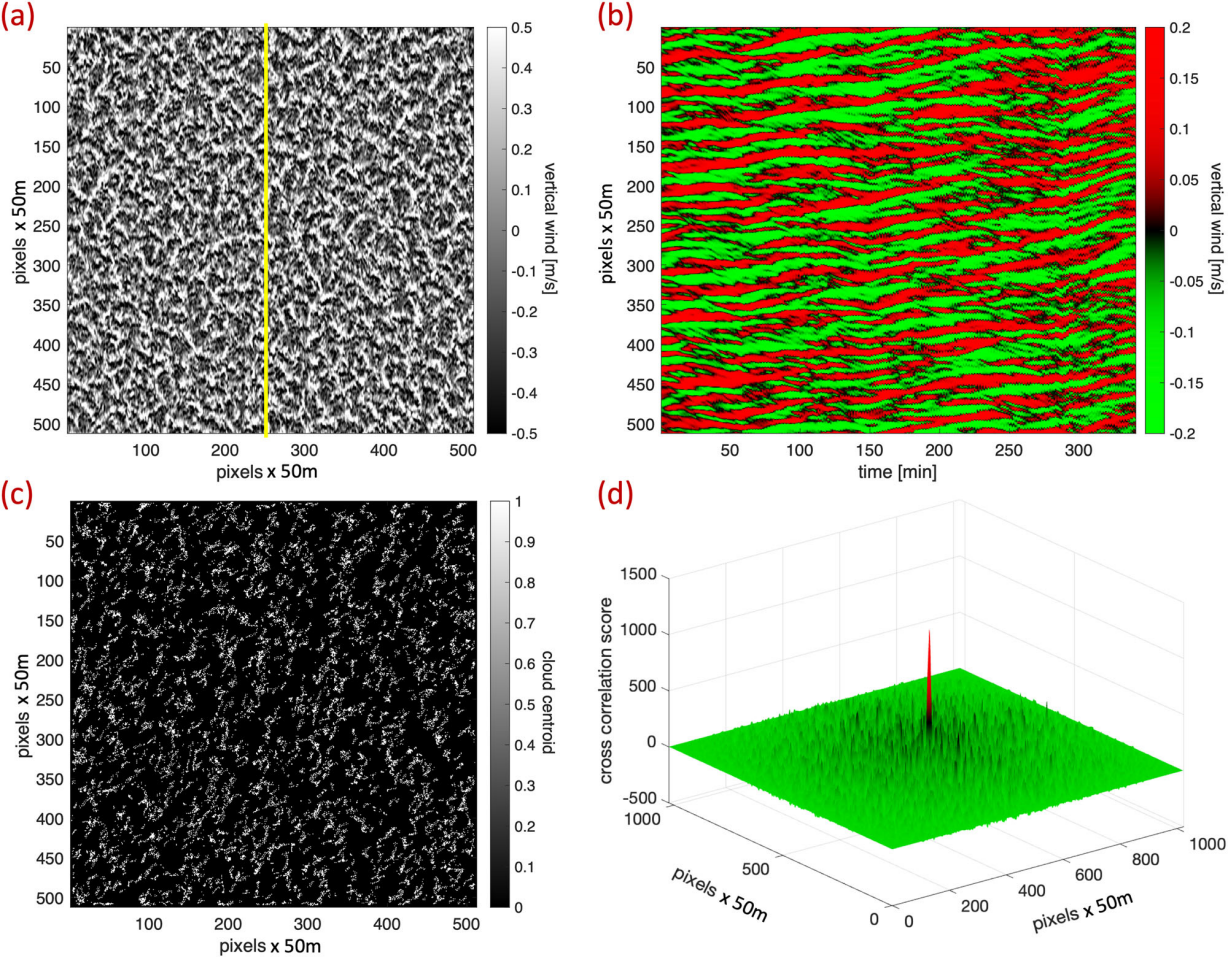
The steady dynamics of dense convective cells allow predicting Trade Cumulus clouds’ location. **a** Snapshot of the vertical velocity below the cloud base (average of the lowest 400 m), showing a field densely occupied with convective cells. Updrafts form along the cell walls, while downdrafts form in the center. The yellow line indicates the location of the vertical trace used to form the *w*-LHS shown in (**b**). **b** The *w*-LHS, a measure of the steadiness of the convective cells, where red stripes indicate updrafts and green downdrafts. The lifetime of many cells is comparable to that of the entire field (6 h). To demonstrate the predictive power of the stable dynamics, (**c**) shows cloud centroids accumulated over 3 h after the time of the snapshot in (**a**). Note the non-random cell structure of the forming clouds. **d** Cross-correlation between (**a**) and (**c**) reveals the similarity between the *w*-field and the clouds forming later. The correlation peak value in the center of the matrix exceeds 20*σ* of the correlation distribution, and the background correlation is almost isotropic.

How similar are these two fields (as seen in Fig. 2a, c)? While visual inspection shows similarities, to gain quantitative insights, we calculated the cross-correlation score of the two normalized fields. Figure 2d displays the 2D cross-correlation space for all possible translations of the cloud centroid field over the updraft snapshot. The center of the correlation space marks the zero translation point, with a notable high score at the center that exceeds 20*σ* of the correlation distribution and rapidly decaying correlations away from it.

Highly regular cloud patterns, such as open and closed Sc cloud fields and marine or continental cloud streets, have recently been shown to emerge from a Convective Steady-State (CSS)^4^. CSS refers to densely packed, thermally driven convective cells continuously dividing the field into updraft and downdraft areas, producing regular and steady cloud patterns. In this study, we analyze the sugar-type trade cumulus (TrCu) clouds, which are regarded as the least organized form of convective clouds. Although coherent structures like streets or cells are sometimes observed in sugar-type TrCu clouds in satellite data^9^, and in simulations^20,21^, these clouds were generally thought to form through localized processes driven by thermal or humidity perturbations that generate buoyant parcels or plumes.

Here, we demonstrate that, like the cloud types forming the most regular patterns, sugar-type TrCu cloud fields also conceal a well-organized structure of steady, thermally driven convective cells densely occupying the entire domain. These convective cells connect the sub-cloud layer with the cloud layer, as indicated by the correlation between the updraft field below the cloud base and the clouds themselves (see Fig. 2d). These cells act as the dynamic building blocks of the cloud field. Notably, the characteristic lifetimes of the convective cells are significantly longer than those of the TrCu clouds. The horizontal stripes in the *w*-LHS (Fig. 2b) suggest that their lifetimes nearly match that of the entire cloud field. Moreover, we show that the steady structure of these cells, both in time and space, can predict the locations of future clouds forming over the updraft regions. The integrated view (Fig. 2c) reveals a cellular structure that aligns with the updraft field (Fig. 2a). These results shift our understanding of such cloud fields from a model where random, localized perturbations create isolated clouds to a more deterministic framework.

Sugar-type TrCu clouds cover large areas over the globe. They play an important climate role, and their overall effect on the radiative energy budget and their feedback to global warming are poorly understood. The new deterministic view of the formation of such clouds should provide new insights into these important questions.

## METHODS

### GOES–16 ABI images

To study the fine patterns of small-sized sugar TrCu clouds, we used the Advanced Baseline Imager (ABI) on board GOES–16. Specifically, we utilized the level 1B “Red” band (channel 2, 0.64 μm) radiance product^12^, which provides high spatiotemporal resolution images (0.5 km in space and 5-min in time over the continental US: CONUS domain).

The radiance values were converted to reflectance following the methodology of ref. ^22^, which applied a simple gamma correction to adjust and brighten the images, and re-projected the images into a geographic (latitude–longitude) projection.

Our focus was on a region over the Caribbean Sea, characterized by a steady easterly flow associated with the trade winds. This region, located between 16°*N*–23°*N* and 76°*W*–86°*W*, was observed on February 6, 2018, a typical day of sugar-type TrCu clouds.

### Model simulations

To investigate the dynamics of TrCu cloud fields, we simulated such a field using the System for Atmospheric Modeling (SAM), an LES model that resolves boundary layer clouds in a nonhydrostatic, anelastic way. The Barbados Oceanographic and Meteorological Experiment (BOMEX) case study of sugar-type TrCu clouds was simulated based on observations made east of Barbados in June 1969^23^. BOMEX observations represent reliable and robust measurements of sugar-type TrCu clouds^2,24^.

The simulation was initialized by surface fluxes and profiles of temperature, humidity, and horizontal wind shear. The model ran with periodic boundary conditions over a domain size of 25.5 km horizontally, with a resolution of 50 m, and 4 km vertically, with a resolution of 40 m. The simulation total time was 8 h, in time steps of 1 s, with the first 2 h considered the spin-up time, which is therefore not analyzed here. The LES model was coupled with a SBM scheme^14^ that solves cloud warm processes explicitly, including droplet nucleation, diffusional growth, collision coalescence, sedimentation, and breakup. The aerosol concentration was initialized to 500 per cm^3^ to ensure non-precipitating sugar-type TrCu clouds.

We explored the time evolution of key dynamical parameters such as updrafts, buoyancy, and liquid water content at various levels within the boundary layer (from the surface up to 1600 m). All of them showed the emergence of organized convective cells. We presented the updraft below the cloud base to demonstrate the emergence of steady convective cells on the sub-cloud layer. To show that the convective system couples the lower boundary layer with the cloudy layer above, we traced the cloud locations and demonstrated that after the Lagrangian correction, they correlate strongly with the lower cellular structures (Fig. 2).

### Lagrangian correction: the tracking algorithm

The high spatiotemporal resolution of the observations and model output allowed us to track the clouds and convection cells as they are advected and examine their evolution using a Lagrangian perspective. The Lagrangian correction algorithm has been described and applied to satellite data^7,25^ as well as to cloud-resolving models^4^. At each time step, the displacement is determined by optimizing the similarity between two consecutive images. The main assumption is that the morphological properties of the selected field change slower than the time interval between two successive snapshots. In the case of the satellite dataset, where we have 2D data (no vertical dimension), we follow the evolution of sugar TrCu clouds by tracking the corrected reflectance. In the modeling case, we track the vertical wind field of the lower marine boundary layer, averaged over a 400 m layer above the ocean surface. At each time step (denoted as time-step index *i*), the corresponding 2D matrix of the corrected reflectance (for observations) or the averaged vertical velocity of the lower 400 m (from the model’s output) is compared to the 2D matrix of the next time step (*i* + 1). The optimal displacement (*r*_*x*_, *r*_*y*_) is determined as the one that maximizes the similarity between the matrices. Subsequently, the entire dataset from time step *i* + 1 onward is shifted by the optimal displacement. The same analysis is then repeated by finding the optimal displacement between the 2D matrix of *i* + 1 and the next time step (*i* + 2). It is worth noting that, similar to the observations where reflectance in each snapshot may originate from scattering across multiple layers, the averaging of vertical velocities does not account for wind shear between horizontal layers. A perfect Lagrangian correction would result in a perfect horizontal line on the LHS. However, horizontal velocities-and consequently, the corresponding displacements of each segment between two time steps-are not always uniform within the selected domain. As a result, some segments may deviate from the ideal horizontal structure. Additionally, imperfections in the corrections can arise from displacements that are not integer multiples of pixels. In such cases, the missing fractions, which are often consistent, contribute to deviations from the horizontal lines.

### Accumulated cloud centroid matrix

To explore the predictive skill of the stable, long-lived convective cells that occupy the boundary layer, as shown in Fig. 2a, b, we create a map with the centroid location of each cloud marked as a dot. To do so, we take the cloud liquid water path (LWP) field, which is the vertically integrated amount of liquid water per pixel, and apply the same Lagrangian correction as we do for the vertical velocity field. Then, for each Lagrangian corrected LWP snapshot, we label all the clouds with LWP > 0.01g/m^2^ and find the coordinates of their centroids. Fig. 2c shows a binary matrix marking (in white dots) the location of all of the clouds’ centroids that formed in the 3-h interval starting from the time of the vertical velocity snapshot shown in Fig. 2a. The similarity between the vertical velocity snapshot and the clouds, that formed in the 3 h after it, is measured by the cross-correlation matrix shown in Fig. 2d.

## Data Availability

All GOES-16 satellite data used in this study are publicly available at https://www.ngdc.noaa.gov/. The Lagrangian corrected data of the simulated cloud LWP, and vertical velocities are available in an online repository at 10.34933/c2ff596a-eb3b-4f77-882d-b633ef971ce5.
